# Morphomolecular identification of heavy parasitic typhlitis in layer flocks: tissue response and cell-mediated reaction

**DOI:** 10.1186/s13028-024-00748-8

**Published:** 2024-07-02

**Authors:** Mohamed A. El-Saied, Marwa M. Attia, Marwa A. Ibrahim, Mohamed Elaish, Mohamed R. Mousa

**Affiliations:** 1 Department of Pathology, Faculty of Veterinary Medicine, Cairo University, PO Box 12211, Giza, Egypt; 2 Department of Parasitology, Faculty of Veterinary Medicine, Cairo University, PO Box 12211, Giza, Egypt; 3 Department of Biochemistry and Molecular Biology, Faculty of Veterinary Medicine, Cairo University, PO Box 12211, Giza, Egypt; 4 Department of Poultry Diseases, Faculty of Veterinary Medicine, Cairo University, PO Box 12211, Giza, Egypt

**Keywords:** *Heterakis gallinarum*, Histotropic migration, Immune response, Layer chicken parasites, Molecular diagnosis, Parasitic typhlitis

## Abstract

**Background:**

*Heterakis gallinarum* (*H. gallinarum*) is a common poultry parasite that can be found in the ceca of many gallinaceous bird species, causing minor pathology and reduced weight gain. Most infections go unnoticed in commercial flocks due to the dependence on fecal egg counts, which are prone to false-negative diagnoses. Furthermore, there is a lack of research on gastrointestinal nematodes that use molecular identification methods, which could be essential for rapid diagnosis and developing efficient control approaches. As a result, the study aimed to look at the cause of mortality in layer chickens induced by *H. gallinarum* in Egyptian poultry farms using morphological, ultrastructural, and molecular characterization. Histopathological, immunohistochemical, and cell-mediated immune responses from damaged cecal tissues were also examined.

**Results:**

Seventy bird samples from ten-layer flocks of different breeds (Native, white, and brown layers) suffering from diarrhea, decreased egg output, and emaciation were collected. Cecal samples were collected from affected and non-affected birds and were examined for parasitic diseases using light and a scanning electron microscope. The mitochondrial cytochrome oxidase 1 (COX1) gene was used to characterize *H. gallinarum*. Our results showed that the collected nematodal worms were identified as *H. gallinarum* (male and female), further confirmed by COX1 gene amplification and sequence alignment. Gene expression analysis of the inflammatory markers in infected tissues showed a significant up-regulation of IL-2, IFN-γ, TLR-4, and IL-1β and a significant down-regulation of the anti-inflammatory IL-10. The mRNA level of the apoptotic cas-3 revealed apoptotic activity among the *H. gallinarum* samples compared to the control group.

**Conclusions:**

Our results implemented the use of molecular methods for the diagnosis of *Heterakis*, and this is the first report showing the tissue immune response following infection in layers: upregulation of IL-1β, IFN-γ, Il-2, and TLR-4, while down-regulation of anti-inflammatory IL-10 in cecal tissue, Cas-3 apoptotic activity and Nuclear factor-κB (NF-κB)activity with immunophenotyping of T-cells in *Heterakis* infected tissue.

## Background

*Heterakis* are small nematodes belonging to the family Heterakidae. Three *Heterakis* species have been detected in domestic birds, identified, and characterized (*H. gallinarum*, *H. dispar*, and *H. isolonche*). The size and shape of the male spicules are the only morphological features that set these species apart from one another [1]. *Heterakis gallinarum* is found in waterfowl and gallinaceous birds such as chicken, turkey, and quail. *Heterakis dispar* is detected in geese and ducks, while *H. isolonche* is prevalent in ducks and identified in turkey, goose, prairie fowl, and quail [2]. *Heterakis* has a direct life cycle in which the eggs are ingested by the host directly. Eggs excreted in feces develop into embryos in the environment within two weeks, or birds can be infected by eating earthworms, which identified as a mechanical transport host or paratenic host. The disease mainly affects chickens housed in litter or with pasture soil contact [3, 4].

The parasites can cause gastrointestinal problems, anemia, emaciation, and even death in poultry, resulting in significant economic losses for the poultry industry. Additionally, *H. gallinarum* is a vector for the protozoan *Histomonas meleagridis,* which causes histomoniasis (also known as blackhead disease) that induces massive pathological changes in the liver and the gut resulting in increased host mortality [5]. *Heterakis gallinarum* is a white, small, round cecal worm that causes minimal tissue reaction but, in heavy infection, results in typhlitis accompanied by petechial hemorrhage and caecal mucosa thickening covered with bloody exudate [6, 7].

The prevalence of helminthic parasites has increased in layer farms due to floor housing systems [8, 9]. Many poultry populations are kept in conventional outdoor production systems [10–12]. Previous studies estimated a prevalence of up to 100% of parasitic infections in free-ranging chickens as in Nigeria and South Africa [9, 13].

These nematodes are the most significant gastrointestinal parasites due to their species' variety and widespread geographic distribution [8, 14, 15]. Geographical variation and flock hygienic measures affect the prevalence of *H. gallinarum* [8, 16, 17]. Poor management practices such as high stocking density, inadequate nutrition, climate, and bad litter conditions increased the *H. gallinarum* burden [1, 4]. Additionally, the gold standard for identifying nematode infections in animals, is the microscopic finding of nematode ova in feces, but now it is insufficient for diagnosing parasitic infections in poultry because *H. gallinarum* ova are nearly identical to those of *Ascaridia galli* [18]. Furthermore, due to the diurnal shifting pattern of *H. gallinarum* ova production, fecal egg counts are susceptible to erroneous negative diagnosis [19]. Therefore, when compared to microscopic detection of nematode eggs obtained from fecal samples, molecular diagnostic approaches may provide improved sensitivity and specificity for diagnosing *H. gallinarum* infection.

Limited studies on *H. gallinarum* pathogenesis, genetics, and morphology have been conducted, however, 'immuno-kinetics and cellular response to the infections remain unaddressed. Herein, this study aimed to record the presence of *H. gallinarum* infection in Egyptian poultry farms, with a reference to morphological, ultrastructural, and molecular examination of *H. gallinarum*, in addition to histopathological, immunohistochemical, and immune response characterization.

## Methods

### Case history; study areas and sample collection

The flocks were targeted and sampled because of a problem with *Heterakis.* A total of seventy suspected bird samples were collected in this field study from ten-layer flocks of different breeds (native, white, and brown layers) from two Egyptian governorates (Table 1). Suspected birds aged 181 to 560 days old, which suffered from emaciation, ruffled feathers, general weakness, diarrhea, and drop in the egg production. Cecal samples were collected from seven infected birds in each farm (n = 70) and divided into three parts: one maintained in 70% alcohol (worm identification), another frozen for molecular assay, and the third preserved in 10% neutral buffered formalin for further analysis (histopathological investigation and immunohistochemistry). Samples were taken from five uninfected healthy control birds in each flock with a total of fifty control birds, following the identical collection procedure for the negative controls. As well as fecal samples from live birds in all flocks were collected to examine parasitic eggs.

**Table 1:** History and location of samples collected

Number	Province	Flock number and breed	Age	Mortality (last 4 days)	Additional notes
1	Qalyobia	7000 (white egg layer)	517 days	6,7,7,7	• Emaciation with diarrhea • Anthelmintic drug from one month ago
2	Giza	12,100 (native layer)	270 days	12,10,11,12	• Drop in egg production • Old litter (poor in quality and high stocking density)
3	Giza	17,500 (white egg layer)	195 days	8,8,9,10	• High stocking density • Drop in egg production
4	Qalyobia	6000 (baladi)	245 days	8,9,7,9	• Bad litter condition and high humidity • Drop in egg production
5	Qalyobia	7500 (brown egg layer)	349 days	3,2,5,5	• Diarrhea and emaciation
6	Qalyobia	6000 (baladi-native layer)	163 days	6,3,6,6	• High humidity
7	Qalyobia	4500 (white egg layer)	354 days	4,3,5,4	• Bad litter condition • Use of anthelmintic drug from 3 months ago
8	Qalyobia	3500 (white egg layer	560 days	4,7,8,8	• Emaciation with diarrhea • Drop in egg production
9	Giza	6000(Brown egg layer)	210 days	8,6,8,8	• Bad litter condition • Drop in egg production
10	Giza	8000(white egg layer)	181 days	4,6,6,7	• High density, diarrhea

### Morphological identification of H. gallinarum

The collected worms of *H. gallinarum* were rinsed in phosphate-buffered saline (pH 7.2) and stored in 70% alcohol. Then, lactophenol was used for clearing worms before mounting them in gelatin. The characterization of *H. gallinarum* was performed based on morphological features using a light microscope (Olympus CX33, Olympus, Japan), as mentioned previously [20].

### Ultrastructural characterization of Heterakis gallinarum

Determination of *H. gallinarum* ultrastructure was performed through a scanning electron microscope (SEM) as previously described [21]. In brief, worms were repeatedly rinsed in 0.9% saline. The collected worms were fixed with 2.5% glutaraldehyde, dehydrated with an ascending ethanol series, and dried in an Autosamdri-815 (Germany) CO_2_ critical point dryer. In a sputter coater (Spi-Module sputter Coater, UK), the adult was coated with 20 nm gold. Fifty specimens (5 worms from each flock) were imaged using a scanning electron microscope (JSM 5200, Electron prob; Microanalyzer Jeol, Japan) at the Faculty of Agriculture, Cairo University.

### Molecular identification and sequencing of Heterakis gallinarum

The worm genomic DNA was obtained using a Wizard Genomic DNA Purification kit (Promega, Madison, WI, USA) from individual *Heterakis* sp*.* specimens (n = 10 representing each farm). The polymerase chain reaction (PCR), which was conducted in a 25µL reaction mix containing 1 µL of template, 1 µL of MyTaq DNA polymerase (Bioline, Meridian Life Science, Memphis, TN, USA), 5 µL of 5X MyTaq buffer (10 pmol of each primer), and dd H_2_O, was carried out using the genomic DNA from each sample as a template. A negative control (RNA and DNA-free water) was included in each PCR reaction to avoid any nonspecific contamination. The COX1 gene primer sequence used was COX1-R (5-AGTTCTAATCATAAGGATATTGGGA-3) and COX1-F (5- TTTCATACAGAATAAATATCAGGA-3) [5]. The following PCR thermo-cycling parameters were used: initial denaturation at 94  C for 5 min; 35 cycles of 94  C for 30 s, 49  C for 45 s, and 72  C for 1 min; and finally, a final extension at 72  C for 10 min. Sanger sequencing of amplicons was performed in both directions (forward and reverse) using the same amplification primers. Sequencing reactions were processed on an ABI 3730XL DNA sequencer (Applied Biosystems, Carlsbad, CA, USA). By using Bio-Edit, the unassembled sequence data were modified and examined [22]. The assembled sequences were analyzed and aligned using the BLAST tool found at (https://blast.ncbi.nlm.nih.gov/Blast.cgi) against other pertinent sequences that had been deposited in the GenBank. The GenBank database received the modified COX 1 sequences and assigned accession numbers. With 1000 bootstrap repetitions, MEGA X's maximum likelihood (ML) approach was employed to evaluate the COX1 sequences [23]. The following criteria were applied: Model: maximum composite likelihood; substitutions: transversions + transitions; lineage pattern: homogenous; rate of variation among sites: uniform. *Trichostrongylus vitrinus* was used as an outgroup.

### Gene Expression analysis

The RNeasy mini extraction kit (Qiagen) was used to isolate the total RNA from the five pooled *H. gallinarum* infected cecal tissue samples from each of the ten farms (n = 50) in line with the manufacturer's recommendations. Both concentration and purity were determined using spectrophotometry at 260 nm to select pure samples based on the 260:280 ratio. Subsequently, the complementary DNA (cDNA) was made using a Revert Aid First Strand cDNA Synthesis Kit (Thermo-scientific, MA, USA) in accordance with the manufacturer's directions after the DNA contamination was cleaned up with DNase I (Fermentas, Lithuania). According to previous publications, gallus sequences deposited in the GenBank were used to create primer sets for detecting the target genes' mRNA levels (Table 2). The primers were constructed using the primer3 program. Applied Biosystems ABI Prism Step One Plus Real-Time PCR System was used to perform real-time PCR using SYBR Green PCR Master Mix (Thermo-scientific, MA, USA) to estimate the relative expression of the selected genes. Amplification of the cDNA was performed by 40 cycles composed of: denaturation (95 °C for 30 s), annealing (58 °C for 30 s) and extension (72 °C for 30 s). Samples were tested in duplicates. The reference gene β-actin was employed for sample standardization purposes. The expression of genes studied in this work was evaluated using a distinct pool of cDNA derived from five non-infected birds cecal which previously screened for parasites. The expression levels of interleukin-1 beta (IL-1β), interleukin-10 (IL-10), Interferon-gamma (IFN-γ), Intelukin-2 (IL-2), Caspase-3 (cas-3), and Toll receptor 4 (TLR-4) were measured. The 2^ΔΔ^Ct method was used to analyze genetic expression data [24].

**Table 2:** Primer sets for detecting the mRNA levels of the target genes

*Gene*	Accession no	Primer sequence (5′→3′)	Tm (^o^C)	Amplicon (bp)	References
*IL-1B*	NM_204524.2	F: GCCTGCAGAAGAAGCCTCG R: GACGGGCTCAAAAACCTCCT	58	203	[51]
*IL-10*	NM_001004414.4	F: ATGGCAGCTTAACGTTCGGT R: ATGGCAAATGCAGAGCCAGA	58	440	[52]
*IL-2*	NM_204153.2	F:TGCAGTGTTACCTGGGAGAAGTGGT R: ACTTCCGGTGTGATTTAGACCCGT	58	140	[53]
*TLR-4*	NM_001030693.2	F: ATGTCCTCTTGCCATCCCAA R: TCTCCCCTTTCTGCAGAGTG	58	158	[51]
*IFN-γ*	NM_205149.2	F: AGCTGACGGTGGACCTATTATT R: GGCTTTGCGCTGGATTC	58	259	[51]
*CAS-3*	NM_204725.1	F: TTGAAGCAGACAGTGGACCA R: GTTCAAGTTTCCTGGCGTGT	58	177	[52]
*β-ACTIN*	NM_205518.1	F: AGAGGCTCCCCTGAACCCCAAAGC R: CTGGATGGCTACATACATGGCTGG	58	94	[52]

### Histopathological examination

The collected ceca were preserved in 10% neutral buffered formalin, then processed by washing in water and dehydrated using ascending grades of alcohol, then cleared with xylene and embedded in paraffin wax and sectioned, stained with hematoxylin and eosin (H&E). The tissue section was examined under a Leica Microscope linked to the Leica camera. The cecal H&E-stained areas were scanned at 200 X using a scanning digital camera (Basler, Germany) connected with a CX33 light microscope (Olympus, Tokyo, Japan).

### Immunohistochemical staining

Immunohistochemistry was conducted on the cecal tissue section for immunophenotyping and inflammatory cell characterization. The paraffin-embedded tissue section was dewaxed in xylene and then dehydrated by descending grades of alcohol. Antigen retrieval was performed by PT link apparatus, and then blocking of endogenous peroxidase was performed; later, the cecal tissue sections were overnight incubated with primary antibodies against Nuclear factor-κB (NF-κB) (sc-8008, Santa Cruz Biotechnology), Caspase-3(sc-7272, Santa Cruz Biotechnology), CD-4 (sc-19641; Santa Cruz Biotechnology) and CD-8 (sc-1181; Santa Cruz Biotechnology) at a dilution of 1:200 were incubated overnight at 4  C in a humid chamber. Afterward, the HRP-labelled detection kit (Bio SB, USA) was used as manufacturer instructions to develop the positive reaction. Negative control slides were obtained by slipping out of the primary antibody stage.

### Statistical analysis

Gene expression data were statistically analyzed using an unpaired t-test using GraphPad Prism version 8.0.0 for Windows after checking for normality and homogeneity of the data, GraphPad Software (San Diego, California, USA). A *P-value* was considered significant when *P* ≤ *0.05*.

## Results

### Gross finding

**Fig. 1:**
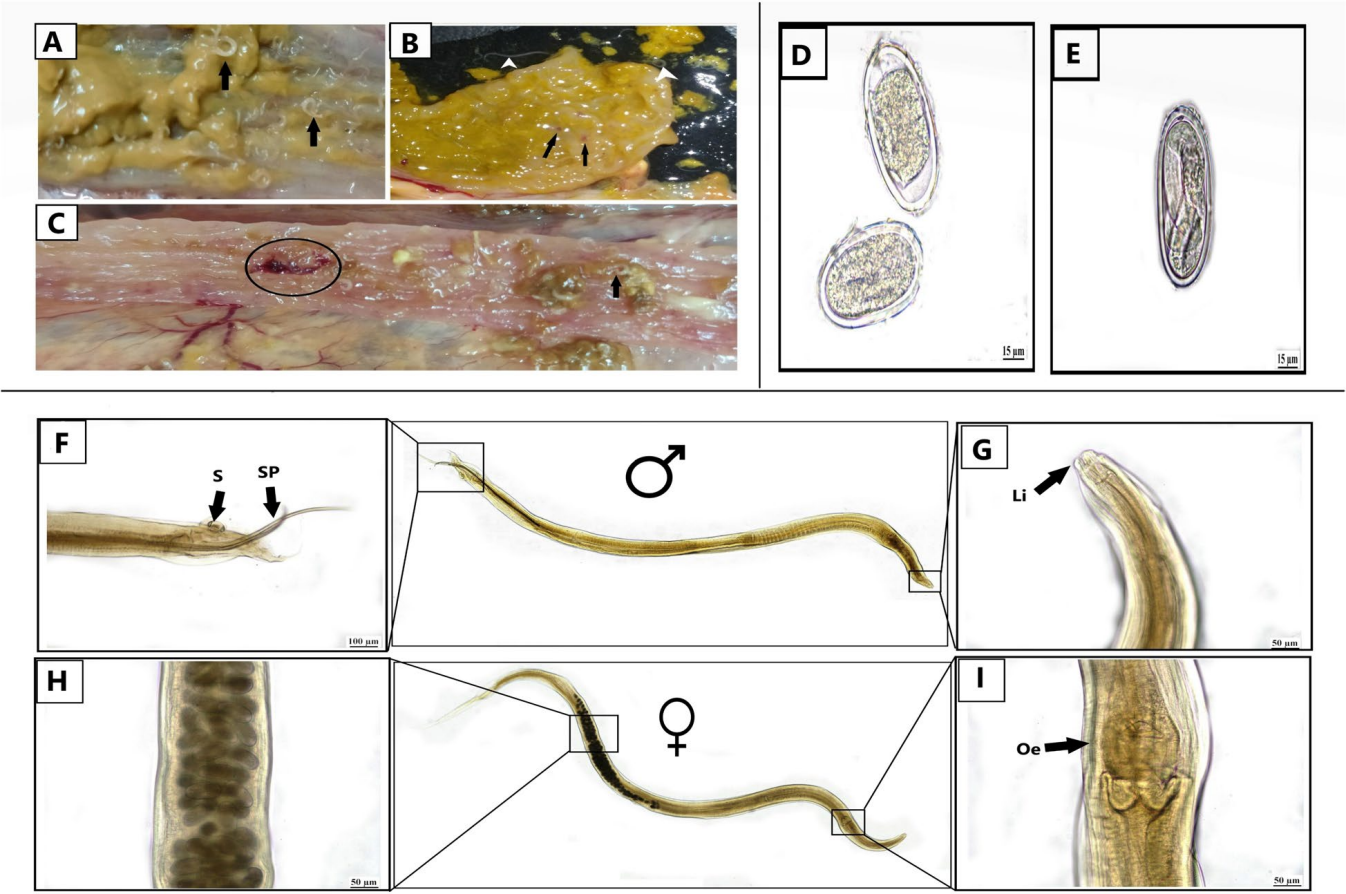
Upper left panel (**A**–**C**): gross finding associated with *H. gallinarum* infection**:** A) The cecal lumen of affected chickens was filled with small slender nematodes of *H. gallinarum* worms (Black arrow), **B** The cecal mucosa was congested and eroded (arrow), **C** Corrugation of the cecal mucosa with hemorrhage (Circle) was noticed beside numerous numbers of *H. gallinarum* worms (Black arrow). Upper right panel (**D**, **E**): *H. gallinarum* D*)* eggs and E) Oval or bean-shaped egg with a smooth, thick shell, containing larva, collected from feces. Lower panel (**F**–**I**): light microscopical micrograph of *H. gallinarum* adult male and female; **F** posterior end of male showing posterior sucker (s) and 2 unequal spicules (sp); **G** anterior end of male and female showing its lips.; **H** middle portion of adult female showing the eggs in the uterus.; **I** the anterior end of male and female with posterior bulb oesophagus

*H. gallinarum* worms were detected in the cecal lumen, the worms were small and slender (Fig. 1A). The cecal mucosa was congested in all examined cases; in some cases, the mucosa was eroded and ulcerated (Fig. 1B). Corrugation of the cecal mucosa and hemorrhage were also noticed (Fig. 1C). The intensity of infection per single chicken was 25–55 (40.6 ± 9.3).

### Morphological identification and ultrastructural characterization of H. gallinarum

**Fig. 2:**
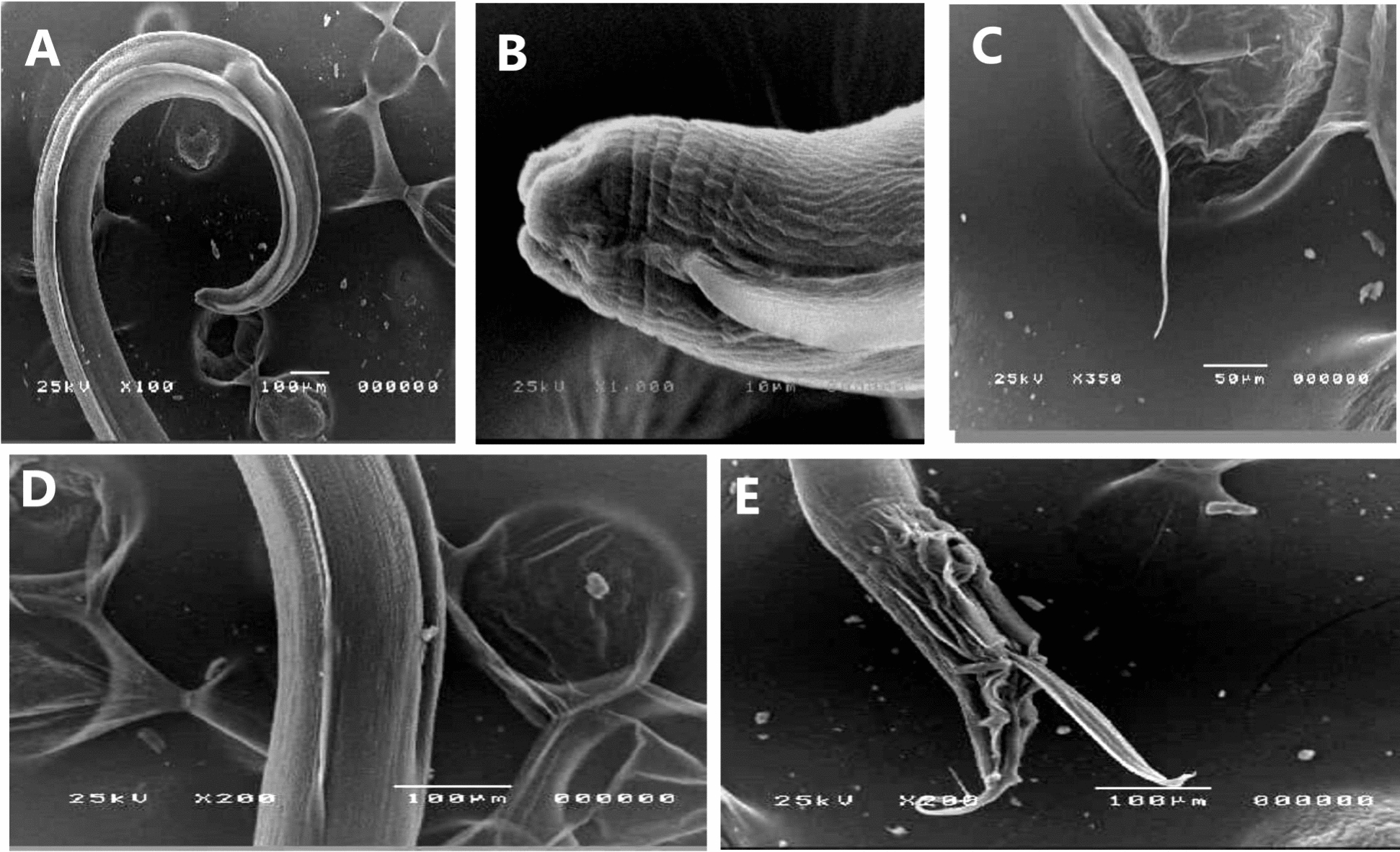
Scanning electron microscopical micrograph of *H. gallinarum* adult male and female; **A**; **B**: anterior end of male and female showing its lips; **C**: posterior end of female showing long tail; **D**: middle portion of adult showing its cuticle, **E**: posterior end of male showing posterior sucker and 2 unequal spicules; with its papillae

Fecal examination and identification were performed to exclude the presence of any other parasites*.* Therefore, the collected nematode worms were identified as *H. gallinarum* male and female; this nematode was medium in size; the female was 13.5 ± 0.619 mm in length, while the male was 10.28 ± 1.49 mm in length. The male posterior end had a circular precloacal sucker measuring 63.33 ± 1.75 µm in length and was surrounded by 12 pairs of papillae. The male had two unequal spicules: the long one measured 1359 ± 95.45 µm, while the short one measured 549.8 ± 3.08 µm. The eggs in the feces were oval or bean-shaped with a thick shell and were smooth, with 69.002 ± 2.19 µm in length and 37.55 ± 0.74 µm in width (Figs. 1 and 2).

### Molecular identification and sequencing of H. gallinarum

The COX1 gene was successfully amplified from the current parasite using the pairs of primers COX1-F and COX1-R [5] and yielded about 894 bp. The sequence was submitted to the GenBank database with the accession number OQ354765. The AT content of this sequence is 65.44%, the GC content is 34.56%, and the average base composition of the sequence is 22.5% (A), 12.4% (C), 22.2% (G), and 42.9% (T). The alignment analysis proved that this parasite belongs to the genus *Heterakis* and has been identified as *H. gallinarum*. The BLAST analysis of the current accession number OQ354765 showed 99.57–97.05% similarity to *H. gallinarum* (ON514033, LC592848, KP308360, OL457533), 87.77–87.56% similarity with that of *H. indica* (LC592875, LC592872), 87.88% similarity to that of *H. beramporia* (LC592867), 87.83% similarity to that of *H. isolonche* (FJ009625), and 86% identity to that of *Ascaridia galli* (KT613900, KT613901).

**Fig. 3:**
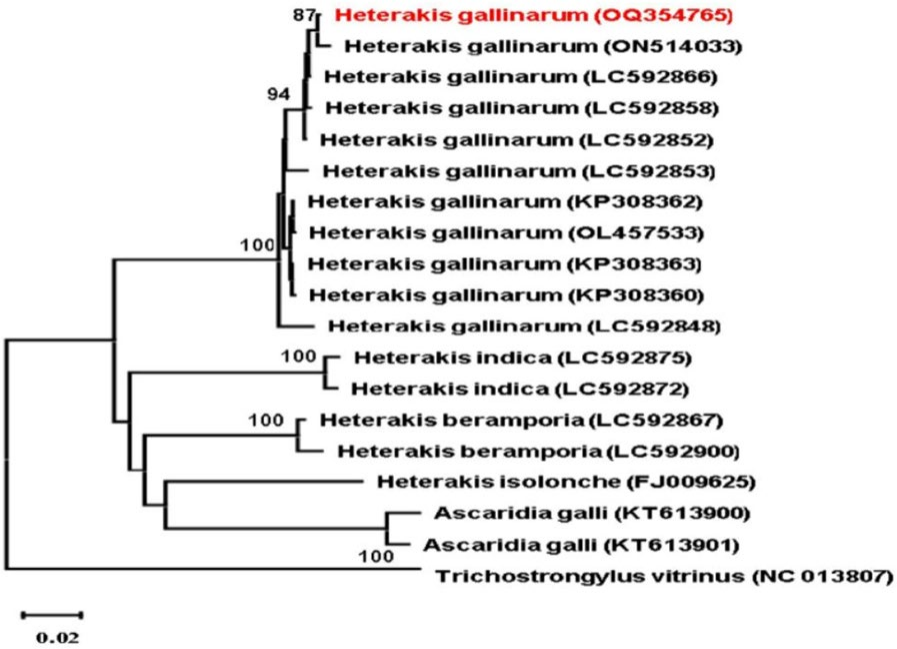
Phylogenetic tree based on the maximum likelihood method using the COX1 gene of *H. gallinarum*

The construction of phylogenetic analysis based on the maximum likelihood method of the COX1 gene revealed two major clades (Fig. 3). All sequences of *H. gallinarum* were grouped with the current sequence to form a monophyletic group in the first clade with 100% bootstrap value. The second clade grouped other sequences of *H. beramporia, H. isolonche, H. indica,* and *Ascaridia galli*.

### Gene Expression analysis

**Fig. 4:**
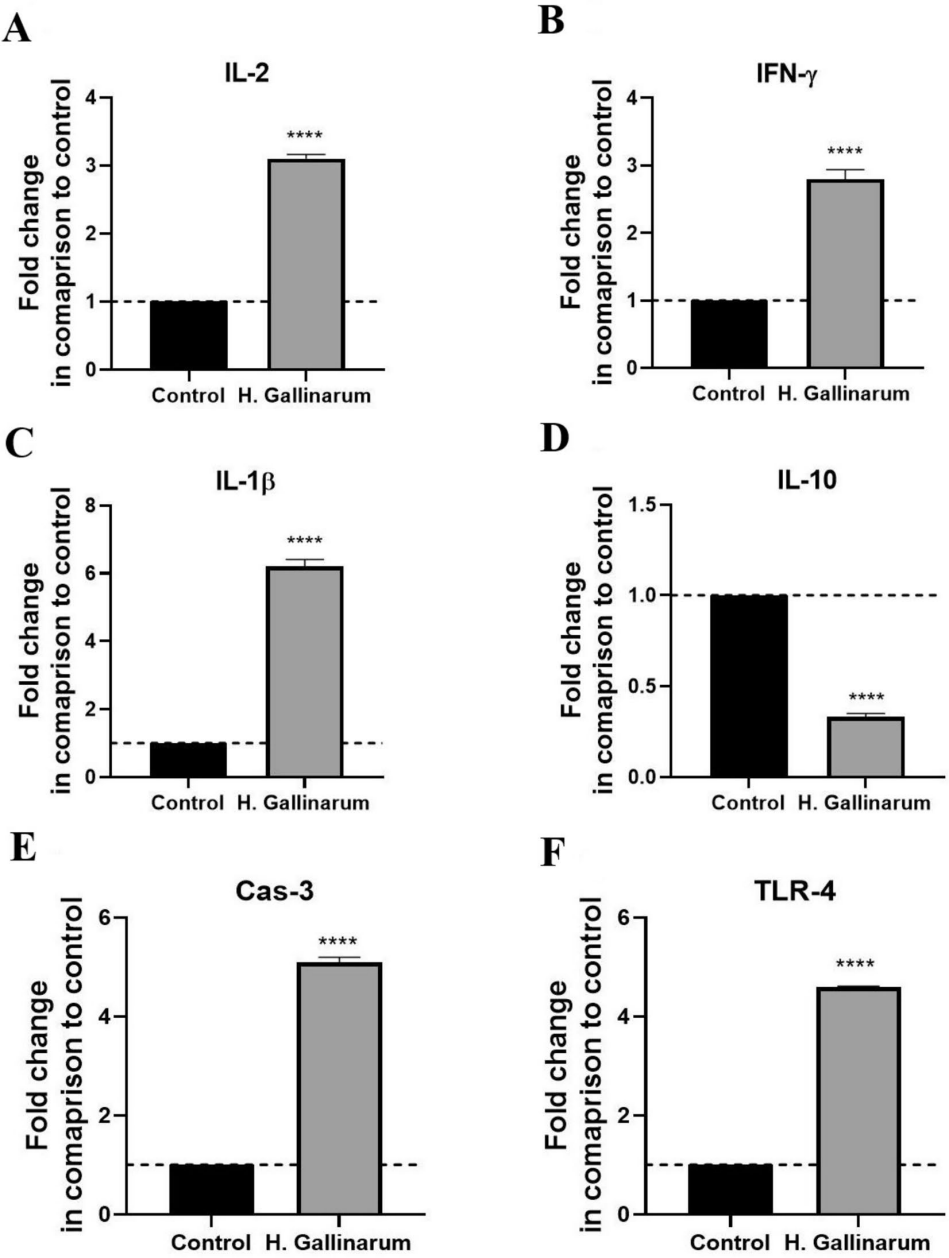
Bar charts representing the transcript levels of **A** IL-2, **B** IFN-γ, **C** IL-1β, **D** IL-10, **E** CAS-3 and **F** TLR4 genes. Values are presented as mean ± SD (n = 50) and * indicates the significant differences to the control group at *P* ≤ *0.05*

Analysis of the transcript level of the inflammatory markers as IL-2, IFN-γ, TLR-4, and IL-1β showed significant upregulation in *H. gallinarum* samples, while there was a significant downregulation of the anti-inflammatory IL-10. Furthermore, the mRNA level of the apoptotic cas-3 revealed apoptotic activity among the *H. gallinarum* samples compared to the control negative (Fig. 4).

### Histopathological changes

**Fig. 5:**
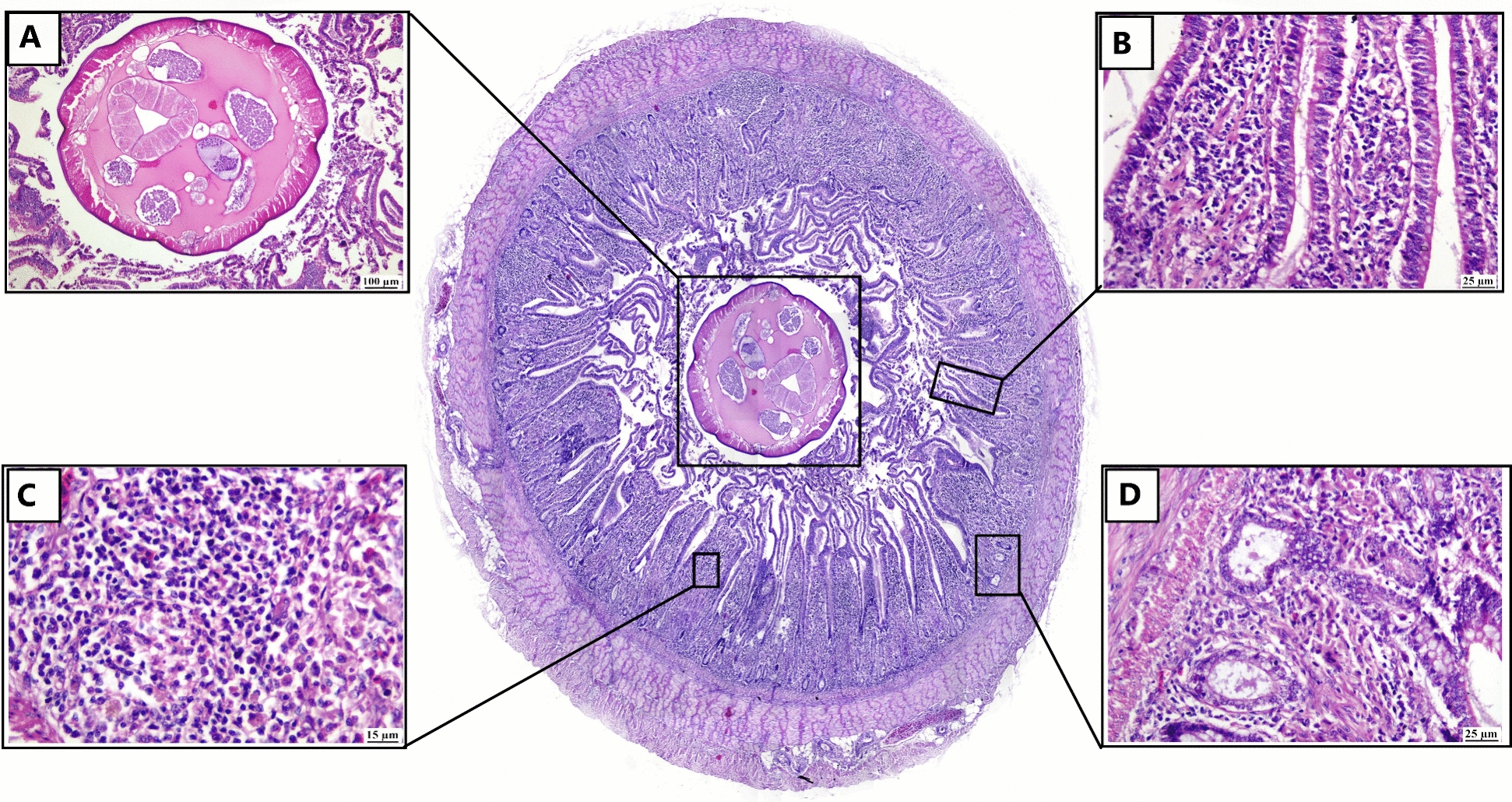
Photomicrograph of intestine of infected bird (H&E). Sub-gross appearance in the center showing occlusion of the intestine lumen with adult *H. gallinarum*. **A** Cross section of adult parasite in the intestinal lumen with eosinophilic smooth cuticle, coelomyarian/polymyarian muscle surrounded by necrotic debris. **B** hyperplasia of goblet cells in the lining epithelium of intestinal villi. **C** lamina propria is infiltrated with numerous mononuclear cells and heterophilic cells. **D** cystic dilated intestinal crypts

**Fig. 6:**
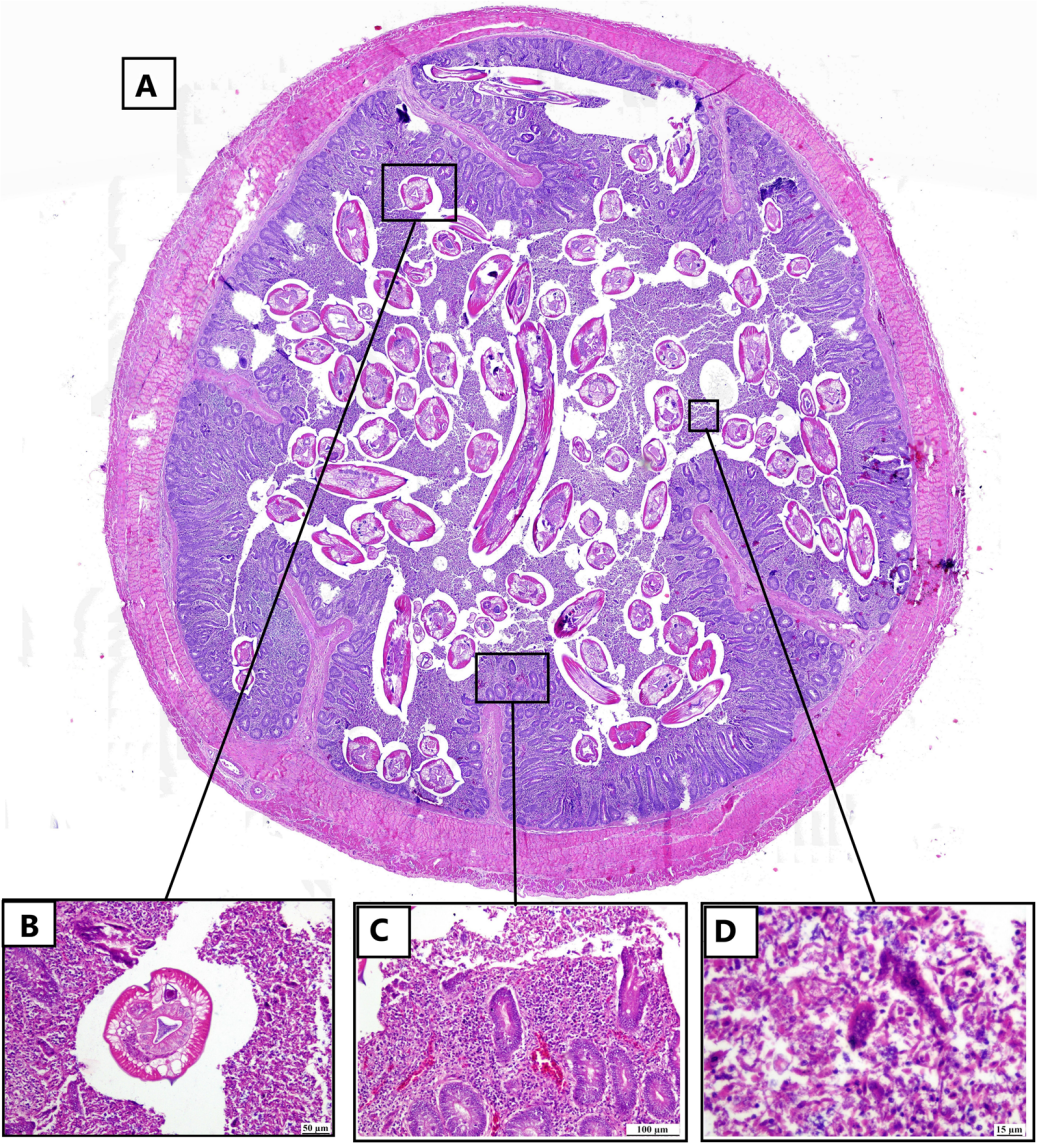
Photomicrograph of intestine of a *H. gallinarum* infected bird (H&E). **A** sub gross appearance of intestine showing heavy infection with *H. gallinarum*. **B** intestinal lumen showing cross section of adult parasite associated with desquamated epithelial cells, inflammatory cells, and necrotic debris. **C** diffuse granulomatous typhlitis in the submucosa with necrosis of mucosal surface. **D** higher magnification showing histiocytic inflammatory cells with giant cells infiltration in cecal lumen

Examined H&E-stained sections revealed parasitic typhlitis with presence of *H. gallinarum* worm (Figs. 5 and 6). Adult cecal nematode obstructed the intestinal lumen (Fig. 5A) that had an eosinophilic smooth cuticle, coelomyarian/polymyarian muscle, lateral alae, pseudocoelom, circular intestinal tract lined with columnar uninucleate cells that have brush border and a uterus containing developing ova. The cecal lining epithelium of some cases was desquamated with goblet cells hyperplasia (Fig. 5B). The lamina propria was heavily infiltrated with lympho-histiocytic cells and giant cells; heterophils were also observed (Fig. 5C). The intestinal crypt exhibited cystic dilation (Fig. 5D). Furthermore, in heavy infection (Fig. 6A), the cecal lumen contained the adult *H. gallinarum* worm with desquamated epithelial cells admixed with mononuclear inflammatory cells. Necrosis of intestinal crypts was also detected (Fig. 6B). Ulcerative and granulomatous typhlitis was noticed, which was characterized by complete epithelial sloughing with mononuclear inflammatory cells infiltration and hemorrhage in the lamina propria (Fig. 6C). Giant cells and histocytes were observed in cecal lumen (Fig. 6D).

### Immunohistochemical staining

**Fig. 7:**
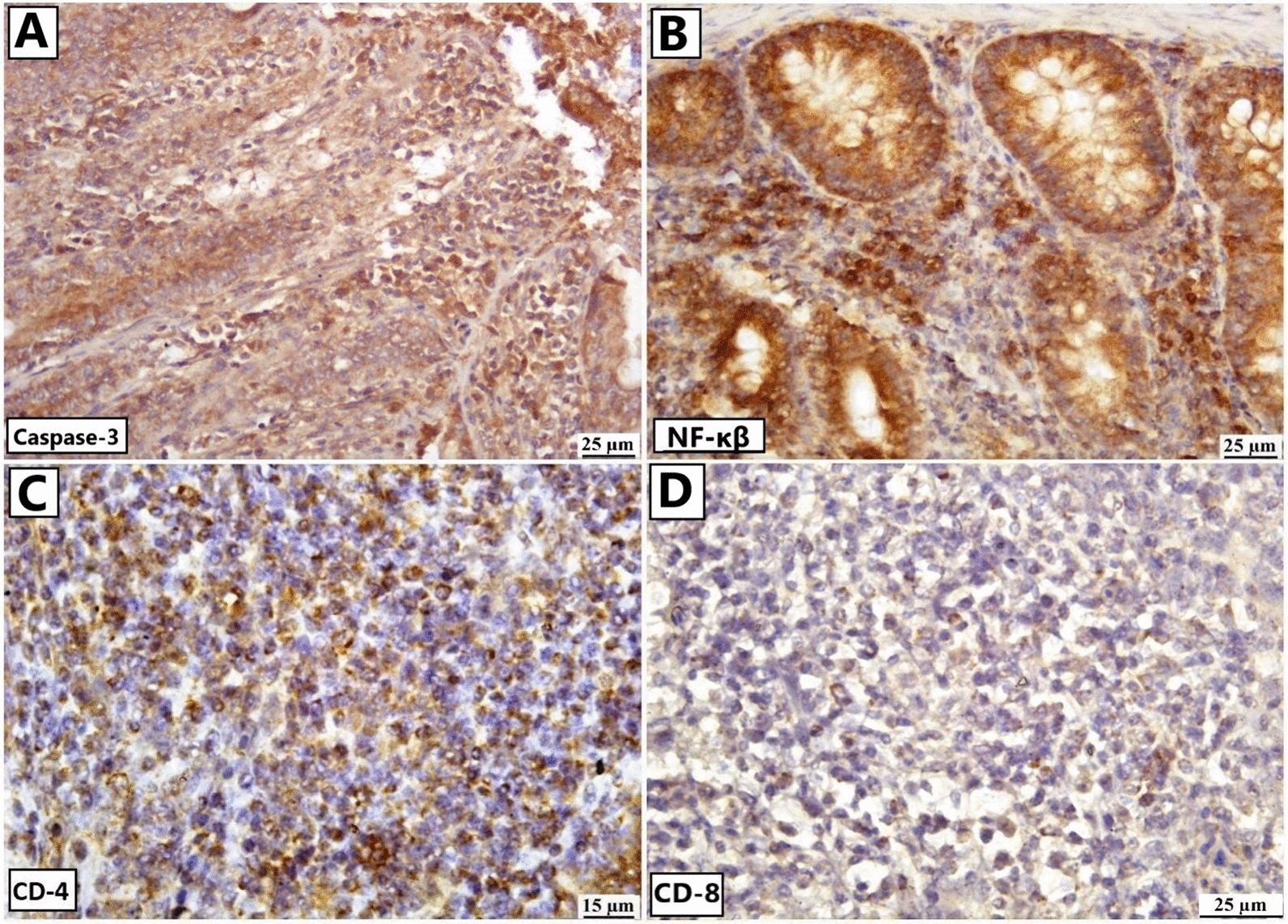
Representative photomicrograph of cecum of *H. gallinarum* infected birds. Immunohistochemical staining (IHC) of intestine showing positive expression of different markers; **A** caspase-3, **B** NF-κb, **C** CD-4 and **D** CD-8

Regarding the immune staining of *Heterakis* infected chickens, positive expression of nuclear factor kappa B (NF-κB) and caspase 3 was detected in both the lining epithelium of intestinal crypt and inflammatory cells in addition to the denuded epithelium. Chiefly, lamina propria was infiltrated with inflammatory cells of CD4 T-lymphocytes and CD8T-lymphocytes (Fig. 7).

## Discussion

*Heterakis gallinarum* has a direct oral-faecal life cycle [25]. The caeca of birds contain adult *H. gallinarum*. When a new host consumes an infectious larvated egg through contaminated food or water, the cycle is completed to form an adult in the large intestine. After hatching in the small intestine, the eggs mature into adults in the caecum (*H. gallinarum*) [26]. The transmission of these nematodes may be facilitated using earthworms as paratenic hosts, but this is not essential [25, 26]. This study described the detection of *H. gallinarum* in Egyptian layer chicken flocks. The presence of nematodes in the caecum confirmed the preferred anatomical sites of *H. gallinarum* as previously mentioned [27–29]. Our study identified the impact of this nematode species as a probable cause of economic losses in poultry production as described before in South Africa, Thailand, Ghana, and Ethiopia [8, 30–33] according to several studies [17, 34–38].

Nematodes are the most prevalent parasitic helminths affecting rural hens, causing financial losses, reduced egg production, weight loss, and increasing the expenses of medical care. Diminished veterinary care of layer chicken predisposes chickens to parasitic diseases. We believe that inadequate husbandry practices at the farm level, as exposure to contaminated litter, could be attributed to the high prevalence of the two nematode species in the hens [35, 39]. The widespread distribution of *H. gallinarum* supports its significance on sub-Saharan African rural chicken production [30, 31, 40–42]. *Heterakis* nematodes represent a frequently encountered parasite in poultry that needs to be controlled [43]. Geographically and among flock management techniques, *H. gallinarum* varies in incidence and typical worm burden. It is challenging to determine the optimal management strategies for lowering *H. gallinarum* infection in chickens because a variety of farm management factors including feed, stocking density, pasture access, pasture rotation, climate, etc.) [30, 31, 40–42]. Poultry raised outdoors are more susceptible to parasite infection, particularly from parasites that have indirect life cycles and need an intermediate host. However, parasite species with fast generation cycles and direct life cycles, like *H. gallinarum*, are more likely to infect chickens maintained in closed houses at high densities.

When it comes to detecting *H. gallinarum* infections, molecular diagnostic methods might be more sensitive and specific than microscopic examination of nematode eggs extracted from faecal samples. Because the ova of *H. gallinarum* and *A. galli* are almost indistinguishable, microscopic examination of nematode eggs in feces—the gold standard for identifying nematode infections in livestock—is insufficient for diagnosing *H. gallinarum* infections in chickens. Additionally, because of the diurnal fluctuations in *H. gallinarum* ova production, faecal egg counts can lead to false negative diagnosis. To find out if *H. gallinarum* is present in a poultry facility, the diagnostic PCR offers a sensitive test that can separate *H. gallinarum* from *A. galli*. However, further investigation is required to determine whether faecal samples are a suitable way to diagnose *H. gallinarum* infections in individual birds [30, 31, 40–42].

There has been a growing interest in understanding the role of various immune molecules in the context of intestinal infections in poultry. One such group of molecules is interleukin cytokines, that play a crucial role in regulating immune responses. Interleukin-1 (IL-1) is a pro-inflammatory cytokine produced by various immune cells, including macrophages and monocytes. IL-1 is involved in activating the inflammatory cells, which is a vital component of the innate immune response [44]. The injury of the cecal mucosa and numerous inflammatory cell infiltration detected in several affected flocks is attributed to the presence of migrating larvae of *H. gallinarum* and granuloma formation, as previously described [45]. The observed histopathological alteration of the intestinal mucosa comes in agreement with the pathological picture observed in naturally infected chicken [46]. Infection with *H. gallinarum* resulted in increased infiltration of CD-4 and CD-8 in the lamina propria and submucosa that could be attributed to tissue injuries induced by parasite irritation [7] that can influence the upregulation of interleukin-2 (IL-2) as it plays a pivotal role in the host immune response. IL-2 is produced by activated T cells and stimulates the proliferation and differentiation of T cells and natural killer cells. In the context of poultry intestinal infections, IL-2 has been shown to play a role in the activation of T cells and the development of protective immunity [47].

Additionally, the activated CD4 and CD8 T cells detected in immune-histochemical findings upregulated interferon-gamma (IFN-γ) expression, which leads to activation of macrophages and enhancing the expression of MHC class I and II molecules on antigen-presenting cells. IFN-γ is thought to have a protective role in activating immune cells and enhancing the clearance of pathogens [48]. Vis-à-vis, severe necrotic and ulcerative typhlitis leading to overexpression of nuclear factor kappa b (NF-κB) and caspase 3 markers as they have a crucial role in apoptosis and inflammation. As a master regulator of the inflammatory response in the intricate inflammatory network, nuclear factor kappa b (NF-κB) is essential for the host's defence against pathogens and prevents cell death by inducing the overexpression of proinflammatory genes. In addition, caspase 3 activation have been linked NF-κB. Also, caspase 3 is activated in response to various stimuli such as oxidative stress and DNA damage. In the context of poultry intestinal infections, caspase 3 is concerned with regulating epithelial cell turnover and the clearance of infected cells [44]. Likewise, Toll-like receptors (TLRs) are a family of receptors that play a critical role in the recognition of pathogens by the innate immune system that increased in *H. gallinarum* infection [49–53]. On the other hand, interleukin-10 (IL-10) is considered an anti-inflammatory cytokine that suppresses pro-inflammatory cytokine production and reduces the immune response. IL-10 has been shown to play a protective role in intestinal infections by reducing inflammation and preventing tissue damage [54–56].

## Conclusions

The immune response to *H. gallinarum* infection in layer chickens involves a complex interplay of immune molecules, including interleukins, TLRs, IFN-γ, caspase 3, and nuclear factor kappa b (NF-κB) with immunophenotyping of CD4 and CD8 T-cells in *Heterakis* infected tissue. Understanding the function of these molecules in the context of poultry diseases may provide important insights into developing new strategies for preventing and treating this infection. A highly standardized infection study is proposed in future studies to rule out gene variations, age, immunization, and other factors affecting immune response.

## Data Availability

The datasets used and/or analysed during the current study are presented in the current study and available from the corresponding author on reasonable request.”

## Abbreviations

COX1: Mitochondrial cytochrome oxidase 1
IFN-γ: Interferon gamma (IFN-γ)
NF-κB: Nuclear factor kappa β
IL-1: Interleukin-1
IL-2: Interleukin-2

## Declarations

### Prior publication

Data included in this article have not been published previously.

## References

[1] Cupo KL, Beckstead RB (2019). *Heterakis gallinarum*, the cecal nematode of gallinaceous birds: a critical review. Avian Dis.

[2] Bobrek K, Hildebrand J, Urbanowicz J, Gaweł A (2019). Molecular identification and phylogenetic analysis of *Heterakis dispar* isolated from Geese. Acta Parasitol.

[3] Phiri IK, Phiri AM, A.Chota, Ziela M, Masuku M, J.Monrad. Prevalence and distribution of gastrointestinal helminths and their effects on weight gain in free-range chickens in Central Zambia. Trop Anim Health Prod. 2007;309–315. 10.1007/s11250-007-9021-5 17847826

[4] Kaufmann F, Da G, Sohnrey B, Gauly M (2011). Helminth infections in laying hens kept in organic free range systems in Germany. Livest Sci.

[5] Amor N, Farjallah S, Mohammed OB, Alagaili A, Bahri-Sfar L (2018). Molecular characterization of the nematode *Heterakis gallinarum* (*Ascaridida:Heterakidae*) infecting domestic chickens (*Gallus gallus domesticus*) in Tunisia. Turk J Vet Anim Sci.

[6] Alemu A (2021). Prevalence on *Ascaridia galli* and *Heterakis gallinarum* of local and exotic chickens at Debrezeit poultry farm agricultural research center poultry farm. J Vet Med Res.

[7] Schwarz A, Gauly M, Abel H, Das G, Humburg J, Weiss AT, Breves G, Rautenschlein S (2011). Pathobiology of
* Heterakis gallinarum
* mono-infection and co-infection with
* Histomonas meleagridis
* in layer chickens. Avian Pathol.

[8] Mlondo S, Tembe D, Malatji MP, Khumalo ZTH, Mukaratirwa S (2022). Molecular identification of helminth parasites of the *Heterakidae* and *Ascarididae* families of free-ranging chickens from selected rural communities of KwaZulu-Natal province of South Africa. Poult Sci.

[9] Opara M, Roseline O, Daniels O, Jegede O (2016). Haemoparasitism of local and exotic chickens reared in the Tropical Rainforest Zone of Owerri Nigeria. Alex J Vet Sci.

[10] Mafu JV, Masika PJ (2003). Small-scale broiler production by rural farmers in the Central-Eastern Cape Province of South Africa. J S Afr Vet Assoc.

[11] Mtileni BJ, Muchadeyi FC, Maiwashe A, Chimonyo M, Dzama K (2012). Conservation and utilisation of indigenous chicken genetic resources in Southern Africa. Worlds Poult Sci J.

[12] Mcainsh CV, Kusina J, Madsen J, Nyoni O (2004). Traditional chicken production in Zimbabwe. Worlds Poult Sci J.

[13] Mwale M, Masika PJ (2011). Point prevalence study of gastro-intestinal parasites in village chickens of Centane district. South Africa Afr J agric Res.

[14] Bazh EKA (2013). Molecular identification and phylogenetic analysis of *Heterakis gallinae* from native chickens in Egypt. Parasitol Res.

[15] Gao JF, Hou MR, Wang WF, Gao ZY, Zhang XG, Lu YX, Shi TR (2019). The complete mitochondrial genome of *Heterakis dispar* (*Ascaridida: Heterakidae*). Mitochondr DNA Part B Resour..

[16] Permin A, Esmann JB, Hoj CH, Hove T, Mukaratirwa S (2002). Ecto-, endo- and haemoparasites in free-range chickens in the Goromonzi district in Zimbabwe. Prev Vet Med.

[17] Thapa S, Hinrichsen LK, Brenninkmeyer C, Gunnarsson S, Heerkens JLT, Verwer C, Niebuhr K, Willett A, Grilli G, Thamsborg SM (2015). Prevalence and magnitude of helminth infections in organic laying hens (*Gallus gallus domesticus*) across Europe. Vet Parasitol.

[18] Daş G, Hennies M, Sohnrey B, Rahimian S, Wongrak K, Stehr M, Gauly M (2017). A comprehensive evaluation of an ELISA for the diagnosis of the two most common *Ascarids* in chickens using plasma or egg yolks. Parasit Vectors.

[19] Wongrak K, Gauly M, Daş G (2015). Diurnal fluctuations in nematode egg excretion in naturally and in experimentally infected chickens. Vet Parasitol.

[20] Soulsby EJLTA-TT. Helminths, arthropods and protozoa of domesticated animals. In., 7th ed NV-edn. London: Baillière Tindall; 1982.

[21] Salem HM, Khattab MS, Yehia N, El-Hack Mea, El-Saadony MT, Alhimaidi AR, Swelum AA, Attia MM. Morphological and molecular characterization of *Ascaridia Columbae* in the domestic pigeon (*Columba livia domestica*) and the assessment of its immunological responses. Poult Sci. 2022;101:101596. 10.1016/j.psj.2021.101596 PMC8693010 34929441

[22] Hall TA (1999). BioEdit: a user-friendly biological sequence alignment editor and analysis program for Windows 95/98/NT. Nucleic Acids Symp Ser.

[23] Kumar S, Stecher G, Li M, Knyaz C, Tamura K (2018). MEGA X: molecular evolutionary genetics analysis across computing platforms. Mol Biol Evol.

[24] Livak KJ, Schmittgen TD (2001). Analysis of relative gene expression data using real-time quantitative PCR and the 2^− ΔΔC^T method. Methods.

[25] Permin A, Hansen JW. Epidemiology, diagnosis, and control of poultry parasites: Food and Agriculture Organization of the United Nations; Rome. 1998;25.

[26] Belete A, Addis M, Ayele M. Review on major gastrointestinal parasites that affect chickens. J Biol Agri Healthcare. 2016;11–21.

[27] Permin A, Christensen JP, Bisgaard M (2006). Consequences of concurrent *Ascaridia galli* and *Escherichia coli* infections in chickens. Acta Vet Scand.

[28] Ngongeh LA, Ugwuzor EG, Fakae BB (2019). Consequences of concurrent infections with *Ascaridia galli* and *Eimeria* in consequences of concurrent infections with *Ascaridia galli* and *Eimeria* in broiler chickens. Asian J Appl Sci.

[29] Anane A, Dufailu OA, Addy F. *Ascaridia galli* and *Heterakis gallinarum* prevalence and genetic variance of *A.galli* in rural chicken from the northern region, Ghana. Vet Parasitol Reg St. 2022;29:100692. 10.1016/j.vprsr.2022.100692 35256120

[30] Asumang P, Delali JA, Wiafe F, Kamil Z, Balali GI, Afua V, Gobe D, Siaw WN, Pinamang G. Prevalence of gastrointestinal parasites in local and exotic breeds of chickens in Pankrono—Kumasi , Ghana. J Parasitol Res. 2019;5746515 10.1155/2019/5746515 PMC6745123 31565425

[31] Poulsen J, Permin A, Hindsbo O, Yelifari L, Nansen P, Bloch P (2000). Prevalence and distribution of gastro-intestinal helminths and haemoparasites in young scavenging chickens in Upper Eastern Region of Ghana, West Africa. Prev Vet Med.

[32] Ahmed J, Duguma A, Regassa D, Belina D, Jilo R (2017). Gastrointestinal nematode parasites of small ruminants and anthelmintics efficacy test in sheep of Haramaya District, Eastern Ethiopia. Animal Vet Sci.

[33] Wuthijaree K, Lambertz C, Gauly M (2017). Prevalence of gastrointestinal helminth infections in free-range laying hens under mountain farming production conditions. Br Poult Sci.

[34] Silva GS, Romera DM, Fonseca LEC, Meireles M (2016). Helminthic parasites of chickens (*Gallus domesticus*) in different regions of São Paulo state. Brazil Rev Bras Cienc Avic.

[35] El-dakhly KM, El-seify MA, Mohammed ES, Elshahawy IS (2019). Prevalence and distribution pattern of intestinal helminths in chicken and pigeons in Aswan. Upper Egypt Trop Anim Health Prod.

[36] Shah MN, Mansoor A, Draz O (2016). Prevalence and identification of nematodes in chickens from district Charsadda, KPK. Pakistan J Biol Environ Sci.

[37] Ola-fadunsin SD, Uwabujo PI, Sanda IM, Ganiyu IA (2019). Gastrointestinal helminths of intensively managed poultry in Kwara Central, Kwara State, Nigeria: Its diversity, prevalence, intensity and risk factors. Vet World.

[38] Berhe M, Mekibib B, Bsrat A, Atsbaha G. Gastrointestinal helminth parasites of chicken under different management system in Mekelle town, Tigray region, Ethiopia. J Vet Med. 2019;1307582. 10.1155/2019/1307582 PMC6388340 30886870

[39] Reta D (2009). Understanding the role of indigenous chickens during the long walk to food security in Ethiopia. Livest Res Rural Dev.

[40] Mungube EO, Bauni SM, Tenhagen BA, Wamae LW, Nzioka SM, Muhammed L, Nginyi JM (2008). Prevalence of parasites of the local scavenging chickens in a selected semi-arid zone of Eastern Kenya. Trop Anim Health Prod.

[41] Malatji DP, Tsotetsi AM, van Marle-Koster E, Muchadeyi FC (2016). A description of village chicken production systems and prevalence of gastrointestinal parasites: case studies in Limpopo and KwaZulu-Natal provinces of South Africa. Onderstepoort J Vet Res.

[42] Tay LK, Emikpe B, Folitse RD, Jarikre T (2017). Point prevalence and pathology associated with gastrointestinal parasites in local chickens and guinea fowls in Kumasi. Ghana Niger J Parasitol.

[43] Norton RA, Clark FD, Beasley JN (1999). An outbreak of *Histomoniasis* in turkeys infected with a moderate level of *Ascaridia* dissimilis but no *Heterakis gallinarum*. Avian Dis.

[44] Mcintosh A, Meikle LM, Ormsby MJ, Mccormick BA, Christie JM, Brewer JM, Roberts M, Wall M (2017). SipA activation of caspase-3 is a decisive mediator of host cell survival at early stages of *Salmonella enterica* serovar Typhimurium infection. Infect Immun.

[45] Riddell C, Gajadhar A (1988). Cecal and hepatic granulomas in chickens associated with *Heterakis gallinarum* infection. Avian Dis.

[46] Butt Z, Memon SA, Shaikh AA. Pathology of *Heterakis gallinarum* in the ceca of naturally infected chicken (*Gallus domesticus*). Pure Appl Biol. 2021;5(4).

[47] Ross SH, Cantrell DA (2018). Signaling and function of interleukin-2 in T lymphocytes. Annu Rev Immunol.

[48] Alspach E, Lussier DM, Schreiber RD. Cancer Immun. 2019;1–20.

[49] Sameer AS (2021). Toll-like receptors (TLRs): structure, functions, signaling, and role of their polymorphisms in colorectal cancer susceptibility. Biomed Res Int.

[50] Iyer SS, Cheng G (2012). Role of Interleukin 10 transcriptional regulation in inflammation and autoimmune disease. Crit Rev Immunol.

[51] Attia MM, Abdelsalam M, Korany RM, Mahdy OA (2021). Characterization of digenetic trematodes infecting African catfish (*Clarias gariepinus*) based on integrated morphological, molecular, histopathological, and immunological examination. Parasitol Res.

[52] Salem HM, Yehia N, Al-Otaibi S, El-Shehawi AM, Elrys AAME, El-Saadony MT, Attia MM (2022). The prevalence and intensity of external parasites in domestic pigeons (*Columba livia domestica*) in Egypt with special reference to the role of deltamethrin as insecticidal agent. Saudi J Biol Sci.

[53] Salem HM, Khattab MS, Yehia N, El-Hack MEA, El-Saadony MT, Alhimaidi AR, Swelum AA, Attia MM (2021). Al SET. Morphological and molecular characterization of *Ascaridia columbae* in the domestic pigeon (*Columba livia domestica*) and the assessment of its immunological responses. Poult Sci.

[54] Xu H, Lu Y, Li D, Yan C, Jiang Y, Hu Z (2023). Probiotic mediated intestinal microbiota and improved performance, egg quality and ovarian immune function of laying hens at different laying stage. Front Microbiol.

[55] Elhady MA, Khalaf AAA, Ibrahim MA, Hassanen EI, Abdelrahman RE, Noshy PA (2022). Protective effects of *Bacillus subtilis* fermentation extract against ochratoxin A-induced nephrotoxicity and immunotoxicity in broiler chickens. J Vet Res.

[56] Yitbarek A, Rodriguez-Lecompte JC, Echeverry HM, Munyaka P, Barjesteh N, Sharif S (2013). Performance, histomorphology, and toll-like receptor, chemokine, and cytokine profile locally and systemically in broiler chickens fed diets supplemented with yeast-derived macromolecules. Poult Sci.

